# Ssk1p-Independent Activation of Ssk2p Plays an Important Role in the Osmotic Stress Response in *Saccharomyces cerevisiae*: Alternative Activation of Ssk2p in Osmotic Stress

**DOI:** 10.1371/journal.pone.0054867

**Published:** 2013-02-14

**Authors:** Hui Zhi, Leihan Tang, Yiji Xia, Jianhua Zhang

**Affiliations:** 1 Department of Biology, Hong Kong Baptist University, Hong Kong, China; 2 Department of Physics, and Center for Quantitative Systems Biology, Hong Kong Baptist University, Hong Kong, China; 3 School of Life Sciences, The Chinese University of Hong Kong, Hong Kong, China; Beatson Institute for Cancer Research Glasgow, United Kingdom

## Abstract

In *Saccharomyces cerevisiae*, external high osmolarity activates the HOG MAPK pathway, which controls various aspects of osmoregulation. MAPKKK Ssk2 is activated by Ssk1 in the SLN1 branch of the osmoregulatory HOG MAPK pathway under hyperosmotic stress. We observed that Ssk2 can be activated independent of Ssk1 upon osmotic shock by an unidentified mechanism. The domain for the Ssk1p-independent activation was identified to be located between the amino acids 177∼240. This region might be involved in the binding of an unknown regulator to Ssk2 which in turn activates Ssk2p without Ssk1p under hyperosmotic stress. The osmotic stress response through the Ssk1p-independent Ssk2p activation is strong, although its duration is short compared with the Ssk1p-dependent activation. The alternative Ssk2p activation is also important for the salt resistance.

## Introduction

The budding yeast *Saccharomyces cerevisiae* live in highly variable environment, in which the *S. cerevisiae* have to cope with the fluctuating external osmolarity [1]. For instance, yeast cells living in the surface of grape berry may be suddenly exposed to the high sugar levels when the outer layer of grapes cracks [2]. Yeast has developed the HOG (high osmolarity glycerol) pathway to survive and adapt to the osmotic stress [2], [3]. The HOG pathway is one of the most detailed-studied MAPK signaling pathways [2], [4]. MAP kinase cascades, the highly conserved signaling pathways nearly in all eukaryotes, are composed of three sequentially activating kinases: a MAPK kinase kinase (MAPKKK) phosphorylates and activates a MAPK kinase (MAPKK), which then activates a MAPK. The HOG cascade consists of five protein kinases [4]. Three MAPKKKs, Ssk2p, Ssk22p, and Ste11p, activate a single downstream MAPKK, Pbs2p, which activates a single MAP kinase, Hog1p [2], [4].

Previous research identified two independent functionally redundant upstream branches, SHO1 branch [5], [6] and SLN1 branch [7], [8], which converge and finally activate the HOG pathway [5], [7]. In the SHO1 branch, two mucin-like transmembrane proteins Msb2p and Hkr1p [9]sense the osmotic shock and together with membrane-bound small G protein Cdc42p, leading to the activation of the PAK-like kinase Ste20p [6], [10], [11]. The activated Ste20p phosphorylates and activates Ste11p [11], which in turn activates the MAPKK Pbs2p [5], [12]. Furthermore, the activation of Ste11p requires the scaffold protein Ste50p which forms a complex with Ste11p. Both the Cdc42-Ste20 and the Sho1-Pbs2 complexes are on the membrane [6], [13], [14], [15].

The SLN1 branch contains a three-component signaling protein complex composed of Sln1p, Ypd1p, and Ssk1p [7], [16], [17], [18], [19], [20]. This upstream branch is structurally and functionally similar to the two-component or three-component phosphorelay systems in certain bacteria and also in plants and other eukaryotes [19], [21]. Sln1p is a sensor histidine kinase which has two TM domains and a cytoplasmic HK domain [8], [21]. Sln1p is catalytically active under normal condition [21]. In an unstressed environment, the Sln1p autophosphorylates itself and this phosphate is then transferred to Asp1144 at the receiver domain of Sln1p [7], [8], [16], [20]. Subsequently, the phosphate group is transferred to His64 on Ypd1p and further to Asp554 on Ssk1p [7], [16], [20]. Dephosphorylated Ssk1p activates the autophosphorylation and activation of MAPKKKs Ssk2p and Ssk22p [22]. Then the activated Ssk2p and Ssk22p activate the Pbs2p [5], [23].

It has also been found that exposure of *ssk1Δste11Δ* mutants to severe osmolarity (0.5 or 1 M KCl) caused clear induction and repression of most osmoregulated genes [24], which indicated that another input into the MAPKKK Ste11p, Ssk2/Ssk22p or putative activation of Pbs2p may exist [24], [25]. Moreover, previous research suggested that Ssk2p may activate the HOG pathway in the absence of Ssk1p after osmotic shock [26]. Here, our genetic analysis confirms this picture and further demonstrates that MAPKKK Ssk2p in the SLN1 branch can be activated independent of Ssk1p under osmotic stress. We identified an important segment near the N-terminal of Ssk2p that is required for the activation independent of Ssk1p. It is possible that another regulator can bind to the N-terminal segment of Ssk2p and activate the Ssk2p to lead to the activation of the HOG pathway. However, the activation of Ssk22p totally depends on the Ssk1p. We also observed that Ssk2p plays an essential role in salt tolerance. Moreover, the alternative input into the Ssk2p is essential for the salt-resistance.

## Materials and Methods

### Yeast Strains

All yeast mutants used in this work are derivatives of the BY4741 strain (Table 1) [27]. The single mutant strains were purchased from Invitrogen. The double and triple mutant strains were generated in our lab by a PCR-based gene deletion strategy [28], [29].

**Table 1:** Strains used in this study.

Strain	Genotype	Source
BY4741	*MATa his3Δ1 leu2Δ0 met15Δ0 ura3Δ0*	INVITROGEN
H1	*MATa his3Δ1 leu2Δ0 met15Δ0 ura3Δ0 hog1Δ::KanMX*	INVITROGEN
H2	*MATa his3Δ1 leu2Δ0 met15Δ0 ura3Δ0 pbs21Δ::KanMX*	INVITROGEN
H3	*MATa his3Δ1 leu2Δ0 met15Δ0 ura3Δ0 ssk1Δ::KanMX*	INVITROGEN
H4	*MATa his3Δ1 leu2Δ0 met15Δ0 ura3Δ0 ste11Δ::KanMX*	INVITROGEN
H5	*MATa his3Δ1 leu2Δ0 met15Δ0 ura3Δ0 ssk2Δ::KanMX*	INVITROGEN
H6	*MATa his3Δ1 leu2Δ0 met15Δ0 ura3Δ0 ssk22Δ::KanMX*	INVITROGEN
H7	*MATa his3Δ1 leu2Δ0 met15Δ0 ura3Δ0 ste11Δ::KanMX ssk1Δ::natMX*	This study
H8	*MATa his3Δ1 leu2Δ0 met15Δ0 ura3Δ0 ssk2Δ::natMX ste11Δ::KanMX*	This study
H9	*MATa his3Δ1 leu2Δ0 met15Δ0 ura3Δ0 ssk22Δ::natMX ste11Δ::KanMX*	This study
H10	*MATa his3Δ1 leu2Δ0 met15Δ0 ura3Δ0 ssk2Δ::natMX ssk22Δ::KanMX*	This study
H11	*MATa his3Δ1 leu2Δ0 met15Δ0 ura3Δ0 ste11Δ::KanMX ssk2Δ::natMX ssk1Δ:: HIS3*	This study
H12	*MATa his3Δ1 leu2Δ0 met15Δ0 ura3Δ0 ste11Δ::KanMX ssk2Δ::natMX ssk1Δ:: HIS3*	This study
H13	*MATa his3Δ1 leu2Δ0 met15Δ0 ura3Δ0 ste11Δ::KanMX ssk22Δ::natMX ssk1Δ:: HIS3*	This study

### Plasmids

Plasmids are listed in Table 2. Deletion and missense mutants were constructed by PCR-based oligonucleotide mutagenesis, and were verified by nucleotide sequencing.

**Table 2:** Plasmids used in this study.

Plasmid	Description	Source
pFA6-kanMX4	*kanMX4* (G418/Geneticin)	[38]
pFA6a-natMX6	*natMX4* (CloNat)	[38]
pFA6a-His3MX6	*HIS3*	[38]
YCplac111	*LEU2, CEN*	[39]
pP111	*LEU2, CEN,* (derivation from YCplac111)	This study
pP111-SSK2	*SSK2, LEU2, CEN*	This study
pP111-SSK2*Δ^(1–176)^*	*SSK2Δ^(1–176)^, LEU2, CEN*	This study
pP111-SSK2*^Δ(1–240)^*	*SSK2^Δ(1–240)^, LEU2, CEN*	This study

### Media and Buffers

In this work, the following growth media were used: YPD (10 g/liter yeast extract, 20 g/liter tryptone, and 20 g/liter glucose), SC (6.7 g/liter yeast nitrogen base, 20 g/liter glucose, and the appropriate yeast synthetic dropout medium supplements). NaCl, KCl, LiCl and sorbitol were added at various concentrations as indicated. Solid culture was performed on 1.5% agar plates of YPD or YPD with NaCl, KCl, LiCl and sorbitol at the indicated concentration.

Stripping buffer contains 62.5 mM Tris-HCl (pH 6.8), 2% SDS and 0.1 M 2-Mecaptoethanol.

### Western Blotting

Cells (5 ml of culture; OD600 = 0.6) were taken at the indicated time points, and collected by brief centrifugation. Dual phosphorylation of Hog1p was detected with the anti-dually phosphorylated p38 antibody (Cell Signaling). Hog1p was examined with specific antibodies (Santa Cruz). Blot was visualized with ECL (34080 Thermo Scientific) after binding of secondary antibody conjugated with horseradish peroxidase. Stripping of the blots was processed at 55°C for 30 min in the stripping buffer.

### Serial Dilution Assays on Plate

To estimate the contribution of activation of the HOG pathway, it was necessary to complement the western blot assay with the qualitative plate growth assay.

For the dilution assays, cells in exponential phase at a concentration of 1×10^6^ cells/ml were diluted 10-fold a total of four times, and 5 µl of each dilution, including the starting dilution of 1×10^6^ cells/ml, was plated on YPD plates containing various stress-inducing agents.

### Latrunculin Treatment

Cells in exponential phase (OD600∼0.8–1.0) were treated with 100 µM latrunculin B (Lat B, Invitrogen; from stock solution 10 mM in ethanol) for 20 min. The same volume of ethanol was added to the control cell culture.

### Microscopy and Rhodamine-Phalloidin Staining

Rhodamine-phalloidin (Rd-phalloidin) staining of actin in yeast was performed as described [30]. Cells were viewed with a laser scanning confocal microscope (FV1000, OLYMPUS; Zeiss 510). In addition, images were displayed using FLV-ASW.

## Results

### Hog1p can be Phosphorylated and Activated in the *ssk1Δste11Δ* Double Mutant

In the HOG pathway, Ssk1p is considered as the activator of Ssk2p and Ssk22p [20]. Early epistasis analysis placed Ssk2p and Ssk22p upstream of Pbs2p and downstream of Ssk1p [5], [8]. If Ssk1p is the sole activator of the Ssk2p and Ssk22p, the double mutant *ssk1Δste11Δ* should be as osmosensitive as *pbs2Δ* and *hog1Δ* mutants and fail to phosphorylate Hog1p upon osmotic shock. However, some studies have found that expression of most osmoregulated genes are clearly induced or repressed in *ssk1Δste11Δ* mutant under severe osmotic stress (0.5 M KCL and 1.0 M KCL) [24]. The observations provided an interesting possibility that additional inputs into Pbs2 may exist [24], [25].

**Figure 1:**
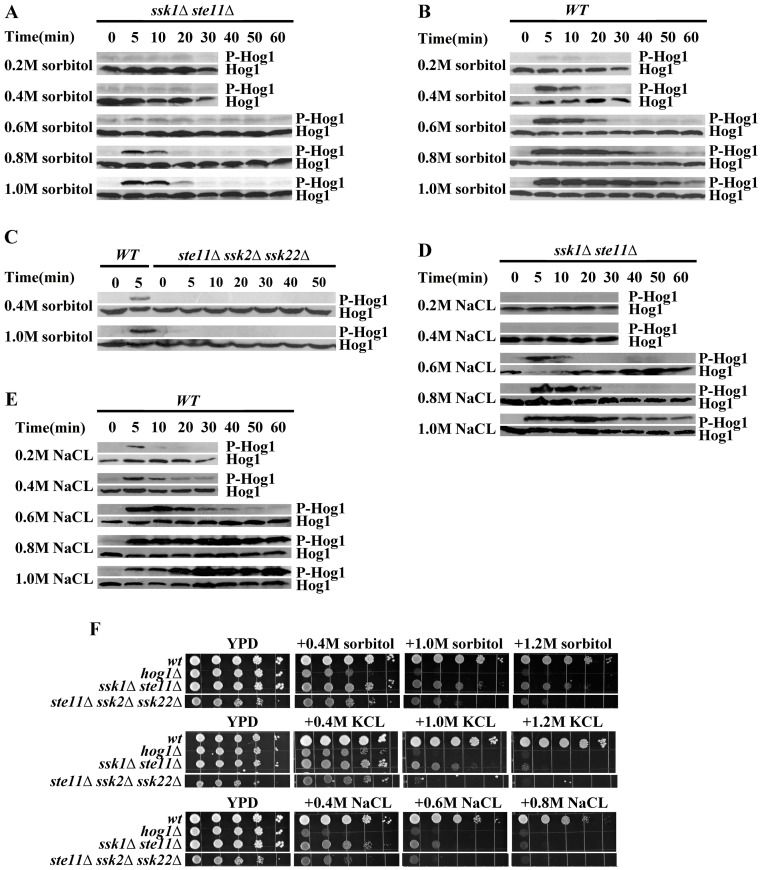
Hog1p phosphorylation level and growth phenotypes for the wild type (WT) and mutant yeast cells under various osmotic and salt stress conditions. A. Hog1p MAPK phosphorylation (P-Hog1p) was detected in the *ssk1Δste11Δ* mutant under hyperosmotic stress. Cells were exposed to different level of osmotic stress induced by sorbitol (concentration shown) in YPD medium for the time indicated. B. Same experiment as in A but for the wild type strain which shows higher sensitivity and a longer duration of the response. C. Hog1p phosphorylation was not detected in the *ste11Δssk2Δssk22Δ* mutant. D. Hog1p phosphorylation assay under ionic osmotic stress in the *ssk1Δste11Δ* double mutant. Cells were exposed to a different levels of salt stress induced by NaCl (concentration shown) in YPD medium for the time indicated. E. Same as in D but for the wild type cells. F. The *ssk1Δste11Δ* mutant exhibited better growth than *hog1Δ* mutant under osmotic stress. Serial dilutions (from left to right in each panel) of indicated strains were spotted onto YPD and salt plates and growth was scored after 3 days.

To identify the alternative pathway, we constructed the double mutant *ssk1Δste11Δ*, and the triple mutant *ste11Δssk2Δssk22Δ*. We carried out the phosphorylation level of Hog1p in the mutant *ssk1Δste11Δ* and *ste11Δssk2Δssk22Δ* under a wide range of osmotic stress conditions (NaCl, KCl and sorbitol, from 0.2 M to 1.0 M). The results, including also measurements on the wide type strain, are shown in Figure 1. We observed that the Hog1p was activated in the *ssk1Δste11Δ* mutant at 0.6 M sorbitol or a higher concentration (Figure 1A). However, Hog1p phosphorylation was not detected under mild osmotic stress (0.2 M and 0.4 M sorbitol/NaCl) in the double mutant (Figure 1A and 1D). In contrast, the phosphorylation of Hog1p could not be detected in the *ste11Δssk2Δssk22Δ* mutant in the wide range concentration of osmotic stress (NaCl, KCl and sorbitol, from 0.2 M to 1.0 M) (Figure 1 C).

Under severe osmotic shock, for instance, 1.0 M sorbitol/NaCl, the phosphorylation of Hog1p peaked within 10 min and lasted for more than 60 min in the wild type strain (Figures 1B and 1E). In the *ssk1Δste11Δ* mutant, although the level of phosphorylation of Hog1p reached was high, the duration was short. In the *ssk1Δste11Δ* mutant, the phosphorylation of Hog1p disappeared within 20 min under 1.0 M sorbitol (Figure 1A). This result is consistent with the transcriptional profiles of osmoregulated genes in the strain *ssk1Δste11Δ* [24]. The expression of several osmoregulated genes (*STL1*, *GRE2*) in *ssk1Δste11Δ* was induced at high level under 0.5 M KCl but the duration of the induction was shorter than that of the wide type strain [24].

Besides, the strain *ssk1Δste11Δ* exhibited much better growth than the *hog1Δ* mutant and the *ste11Δssk2Δssk22Δ* mutant under osmotic stress (Figure 1F). However, the growth of *ssk1Δste11Δ* mutant under osmotic stress depended greatly on the type of osmostressor. The mutant *ssk1Δste11Δ* show better osmoresistance under nonionic osmostressor (sorbitol) (Figure 1 F) than under ionic stress even the Hog1p was similarly phosphorylated under ionic stress. The *ssk1Δste11Δ* cells grew better under KCL stress than under NaCL stress (Figure 1 F).

### Ssk2p can be Activated Independent of Ssk1p under Severe Osmotic Stress

As described above, the HOG pathway was activated in the *ssk1Δste11Δ* mutant under osmotic stress but not in the *ste11Δssk2Δssk22Δ* mutant, which indicated Ssk2p and Ssk22p may be activated independent of Ssk1p under osmotic stress. It has also been reported that the *ssk1Δssk22Δsho1Δ* cells showed better resistance to 500 mM NaCl and 1.5 M sorbitol than *ssk1Δ ssk2Δssk22Δsho1Δ* cells did [26].

**Figure 2:**
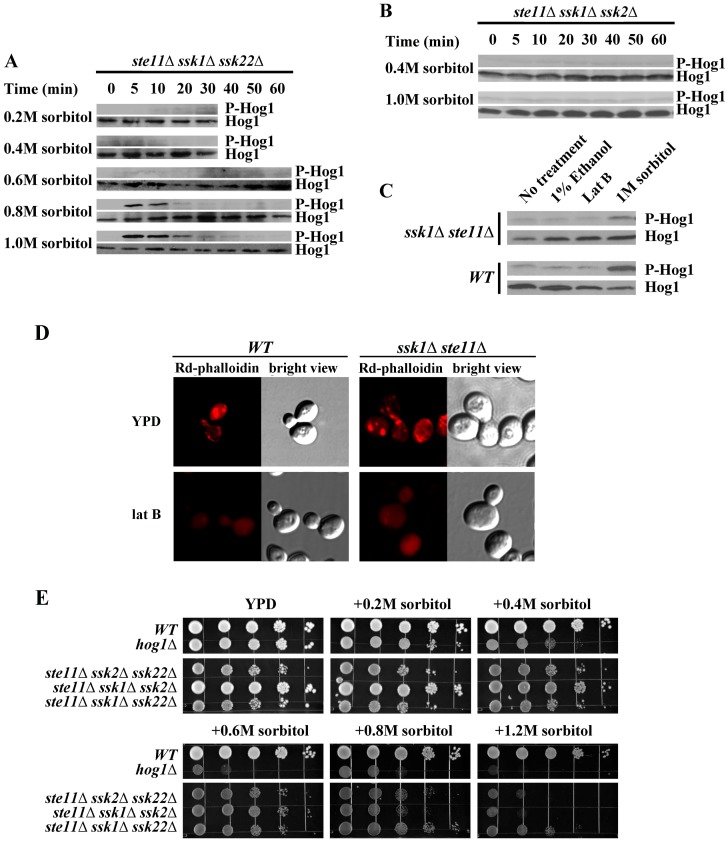
Ssk2p can be activated independent of Ssk1p under severe osmotic stress. A. Hog1p was phosphorylated in the *ste11Δssk1Δssk22Δ* mutant under severe osmotic stress (higher than 0.5 M sorbitol). B. Hog1p could not be phosphorylated in the *ste11Δssk1Δssk2Δ* mutant under 0.4 M or 1.0 M sorbitol. C. Actin disassembly did not activate the HOG pathway through Ssk2p. Within Lat B treatment, wild type strain and *ste11Δssk1Δ* mutant did not display activation of Hog1p. D.The effect of Lat B on actin structures in yeast cells. Rd-phalloidin was used to observe the effects of Lat B addition to yeast cells. Both the wild type cells and *ste11Δssk1Δ* mutant cells were incubated in the absence of Lat B and for 20 min in the presence of 200 mM Lat B. E. The osmosensitivity phenotype of budding yeast HOG pathway mutants. Serial dilutions (from left to right in each panel) of indicated strains were spotted onto YPD and salt plates and growth was scored after 3 days.

To further analyze the alternate activation pathway independent of Ssk1p and Ste11p, we constructed two triple mutants: the *ste11Δssk1Δssk2Δ* mutant and *ste11Δssk1Δssk22Δ* mutant to analyze the phosphorylation state of Hog1p under osmotic stress. Figure 2 shows measurements of the phosphorylation level of Hog1p as well the growth phenotypes in our experiments with the mutant cells. The HOG pathway was activated in the absence of Ste11p, Ssk1p and Ssk22p (Figure 2A) and was inactive if the *STE11*, *SSK1* and *SSK2* were deleted (Figure 2B). The Hog1p was significantly phosphorylated in the *ste11Δssk1Δssk22Δ* mutant under severe osmotic stress (higher than 0.6 M sorbitol). This implies that the MAPKKK Ssk2p can be activated in the absence of Ssk1p under severe osmotic stress. Moderate osmotic stress (concentration lower than 0.4 M sorbitol), on the other hand, could not lead to significant phosphorylation of Hog1p. The phosphorylation pattern of Hog1p under the stress in *ste11Δssk1Δssk22Δ* mutant in Figure 2A is similar to that of the *ssk1Δ ste11Δ* mutant shown in Figure 1A. In *ste11Δssk1Δssk22Δ* mutant, the phosphorylation of Hog1p peaked within 10 min and disappeared within 20 min under 1.0M sorbitol. The duration of the phosphorylated state of Hog1p in *ste11Δssk1Δssk22Δ* mutant was also shorter than wild type (Figure 1B). However, the response to the stress in the *ste11Δssk1Δssk22Δ* mutant was quick. The activation of Ssk22p, on the other hand, was totally dependent on Ssk1p. In *ste11Δssk1Δssk2Δ* mutant, we could not detect any phosphorylation of Hog1p under hyperosmotic stress (Figure 2B). Our results suggest that there may be an unidentified factor that activates Ssk2p under osmotic stress in addition to Ssk1p. Here we name the unidentified factor “X factor” temporarily. The growth of *ste11Δssk1Δssk22Δ* mutant was faster than that of the *ste11Δssk1Δssk2Δ* mutant (Figure 2E).

It has been reported that Ssk2p is specialized to promote actin cytoskeleton reassembly after osmotic shock [31], [32]. This function requires the kinase activity of Ssk2p [26], [31]. Osmotic stress induces a rapid disassembly of the actin cytoskeleton [31], [33]. Actin cytoskeleton disassembly induces Ssk2p to translocate from the cytosol to the septin cytoskeleton of the bud neck [26], [31], [32]. Therefore, we asked whether actin disassembly would activate the Ssk2p to activate the HOG pathway. Lat B was used to induce rapid and complete disassembly of the actin cytoskeleton in strains BY4741 and *ste11Δssk1Δ* [34]. Within 20 min of Lat B treatment, neither strain displayed activation of Hog1p (Figure 2C). After 20 min incubation of both cells in 200 uM lat B, samples were fixed for Rd-phalloidin staining of actin structures. No actin structures were observed in the cells (Figure 2D). The results were in accordance with previous observation that activity of Hog1p activity is affected neither by actin-destabilizing drug latrunculin A, nor by actin-stabilizing drug jasplakinolide [21]. These results indicate that X factor may not be the actin disassembly.

### A Receiver Domain (Amino Acids 177∼240) Near the N-terminus of SSK2 is Needed for the Activation of SSK2 Independent of SSK1

**Figure 3:**
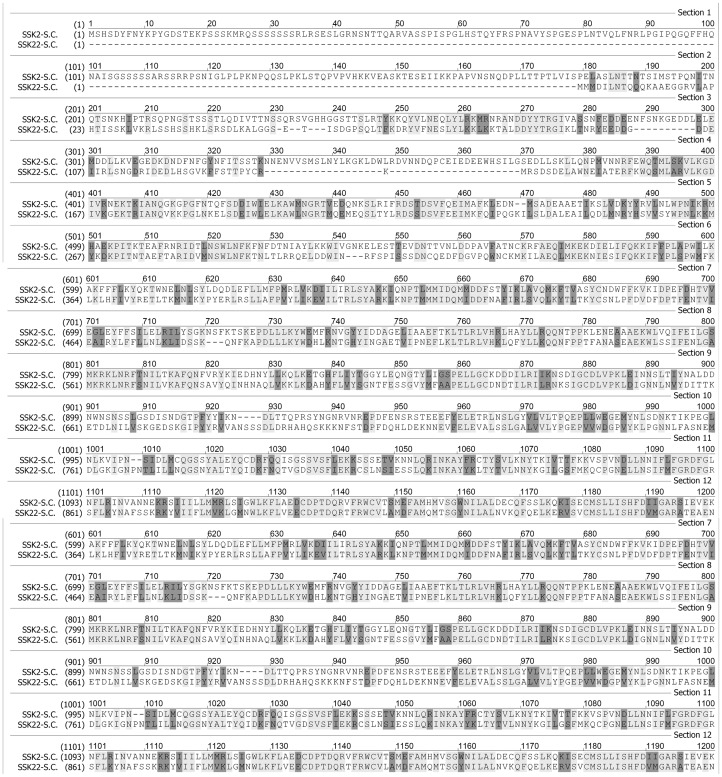
Comparison of protein sequences of Ssk2p and Ssk22p. The alignment was carried out by software Vector NTI 10.

As observed above, Ssk2p can be activated without Ssk1p under osmotic stress, whereas the Ssk22p cannot. We carried out a sequence alignment analysis of the two proteins Ssk2p and Ssk22p. As shown in Figure 3, the sequence comparison shows that Ssk2p and Ssk22p are quite similar. The similarity of the kinase domains of these two MAPKKKs is higher than that of the N-terminal noncatalytical domains. Ssk2p is larger than Ssk22p, mainly due to an extra N-terminal segment (1∼176). There is a high variable N-terminal segment (177∼240) in Ssk22p. Previous study has identified the Ssk1p binding domain (294∼413) in Ssk2p [7]. We assume that the binding site for the X factor is located in the near N-terminal region.

**Figure 4:**
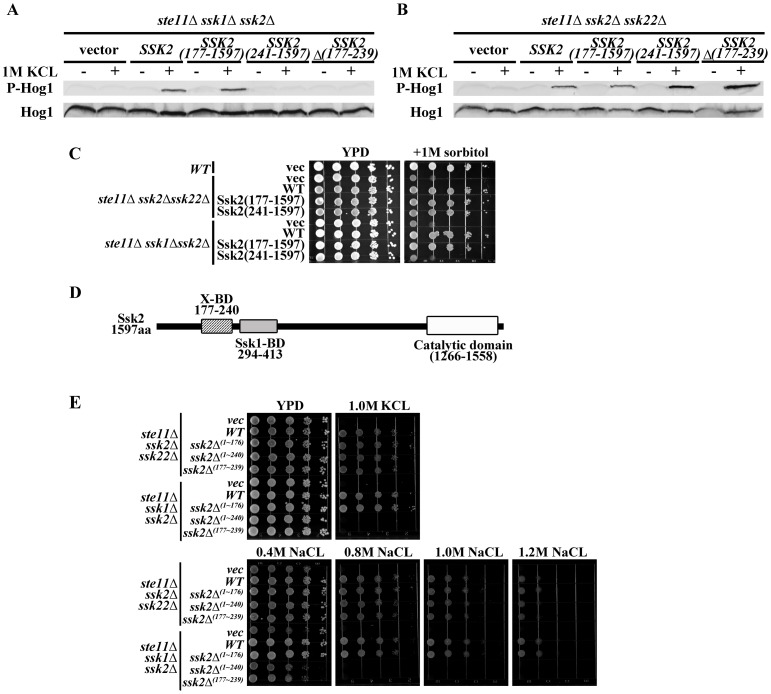
A receiver domain (amino acids 177∼240) near the N-terminus of SSK2 is needed for the activation of SSK2 independent of SSK1. A. Three mutants *ssk2Δ^(1–176)^*, *ssk2Δ^(1–240)^*, *ssk2Δ^(177–239)^* and wild type Ssk2 were expressed in *ste11Δssk1Δssk2Δ* mutant, and the Hog1p phosphorylation was determined under osmotic stress. B. Three mutants *ssk2Δ^(1–176)^, ssk2Δ^(1–240)^, ssk2Δ^(177–239)^* and wild type Ssk2 were expressed in *ste11Δssk2Δssk22Δ* mutant, and the Hog1p phosphorylation was determined under osmotic stress. C. The osmosensitivity of the mutants of Ssk2p. D. N- terminal portion of Ssk2p (amino acid 177–240) is required for the activation of Ssk2p by the X factor under osmotic stress.

To determine the region in Ssk2p that is essential for its activation in the absence of Ssk1p, various segments near the N-terminus were deleted in Ssk2p. These constructs were transformed into the host strains *ste11Δssk2Δssk22Δ* and *ste11Δssk1Δssk2Δ* to test the activation of Hog1p, with results shown in Figures 4A and 4B. A mutant lacking the region (1∼176) was able to activate the Hog1p in both the mutant hosts *ste11Δssk2Δssk22Δ* and *ste11Δssk1Δssk2Δ*. The mutant lacking segment of amino acid 1∼240 could activate the Hog1p in the *ste11Δssk2Δssk22Δ* but not in the host *ste11Δssk1Δssk22Δ*. Besides, the mutant lacking the region of amino acid 177∼239 could activate the HOG pathway in the *ste11Δssk2Δssk22Δ* but not in the host *ste11Δssk1Δssk22Δ*. The phenotype of Ssk2*Δ* ^(177∼239)^ cells is similar to that of the Ssk2*Δ* ^(1∼240)^ cells (Figure 4E). As the growth assay in Figures 4C and 4E show, the *ssk2Δ^(1∼176)^* mutant in both hosts had no discernible effect on growth on high osmolarity media. The *ssk2Δ^(1∼240)^* mutant and *ssk2Δ^(177∼239)^* mutant in *ste11Δssk1Δssk22Δ* were osmosensitive, but not in the *ste11Δssk2Δssk22Δ*.

These results suggest that the N- terminal portion of Ssk2p (amino acids 177–240) is indeed required for the activation of Ssk2p by the X factor under osmotic stress (Figure 4C). The segment is close to the Ssk1p BD (294∼413) (Figure 4 D). Previous research suggested that deletion of the N- terminal region of Ssk2p (amino acids 177–240) would not affect the binding of Ssk2p to either Ssk1p or actin [7], [22], [26].

### Three MAPKKKs Involved in the HOG Pathway have Different Properties

Early research shows that the three MAPKKKs activate the Pbs2p then activate the HOG pathway under osmotic stress [5], [11], [23]. They work functionally redundant to some extent [2], [35], [36]. However, the three MAPKKKs have different activation patterns.

**Figure 5:**
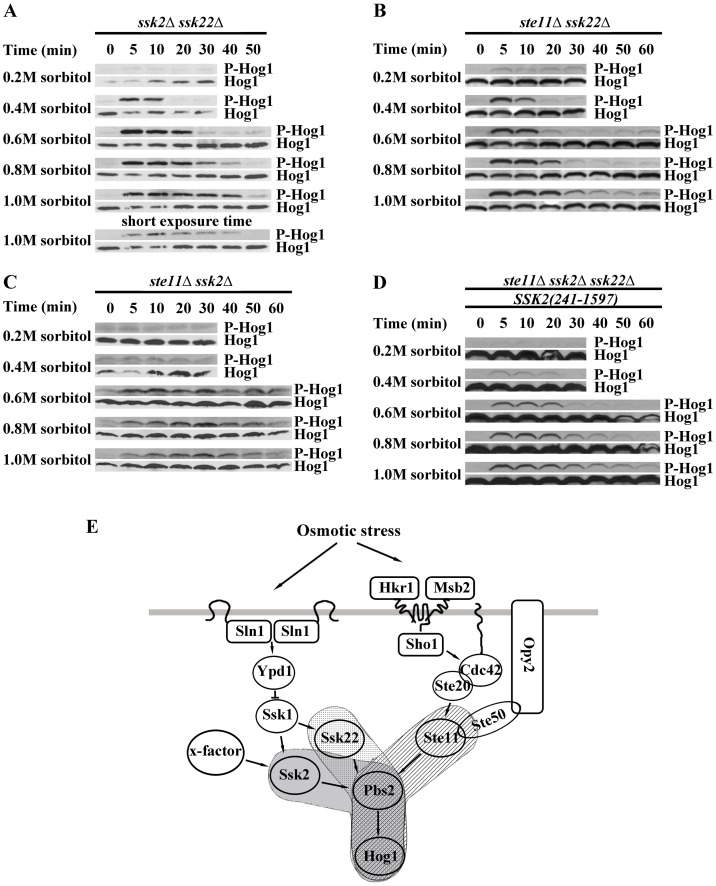
Three MAPKKKs involved in HOG pathway have different properties. A. Hog1p phosphorylation pattern of *ssk2Δssk22Δ* mutant. B. Hog1p phosphorylation pattern of *ssk22Δste11Δ* mutant. C. Hog1p phosphorylation pattern of *ssk2Δste11Δ* mutant. D. Hog1p phosphorylation pattern of *ste11Δssk22Δssk2 Δ^(1–240)^*. E. Scheme of HOG pathway.

To study the activation patterns of the three MAPKKKs, we constructed the double mutant strains: *ssk2Δssk22Δ*, *ste11Δssk2Δ* and *ste11Δssk22Δ* and analyzed the phosphorylation of Hog1p under various levels of stress. Results of our experiments are presented in Figure 5.

In the *ssk2Δssk22Δ* mutant that completely relies on Ste11p for Hog1p activation, the phosphorylation of Hog1p was weakly detected under 0.2 M sorbitol and exhibited a slower maximum response to the severe osmotic stress (1.0 M sorbitol) than the response of the wild type strain (Figure 5A). Under severe osmotic shock (1.0 M sorbitol), the phosphorylation of Hog1p peaked within 5 min in wild type strain (Figure 1B). In the *ssk2Δssk22Δ* mutant, Hog1p phosphorylation peaked at about 10 min under 1.0 M sorbitol.

Cells lacking *STE11* and *SSK2* (*ste11Δssk2Δ* mutant) did not respond to low osmolarity (up to 0.4 M sorbitol), and displayed much slower response at each osmolarity (Generally the peak falls at the 30 minutes point after osmotic shock ), and showed a much lower maximum response than the response of the wild type and other double mutants (Figure 5C). In the *ste11Δssk2Δ* mutant, Hog1p phosphorylation peaked at 30 min under 0.8 M and 1.0 M sorbitol.

In the *ste11Δssk22Δ* mutant which has a functional Ssk2p, cells responded as fast as wild type cells, with the maximum response that was similar to that of the wild type. But the duration of the response of the *ste11Δssk22Δ* mutant under severe osmotic stress (0.8 M–1.0M sorbitol) was shorter than that of the *ssk2Δssk22Δ* mutant and wild type strain (Figure 5B). However, as we discussed above, there are at least two inputs into the Ssk2p: Ssk1p and the X factor (Figure 5E). Therefore, the activation of Hog1p by Ssk2p should be divided into two parts. Pattern of Hog1p’s activation by the X factor can be considered similar to that of the mutant *ste11Δssk1Δssk22Δ* (Figure 2A). To test the activation pattern of Ssk1p, we transformed the mutant Ssk2p lacking the segment of amino acids 1∼240 into the triple mutant *ste11Δssk2Δssk22Δ*. Phosphorylation of Hog1p was detected in the transgenic line under a range of osmotic stress at various time points (Figure 5D). Unexpectedly, the cells expressing the mutant *ssk2Δ (1∼240)* lost the sensitivity to the mild osmotic stress (0.2 M sorbitol) (Figure 5D). Furthermore, the response is significantly attenuated under osmotic stress compared with that of the wild type Ssk2p. The strain *ste11Δssk1Δ* showed a quick response with a high amplitude.

### Ssk2p Plays Essential Role in Salt Tolerance

Previous studies have demonstrated the redundant role of Ssk2p and Ssk22p. Actually, upon nonionic osmotic stress, the Ssk2p and Ssk22p can function equally well. However when subjected to the ionic osmotic stress, the double mutants display different tolerance.

**Figure 6:**
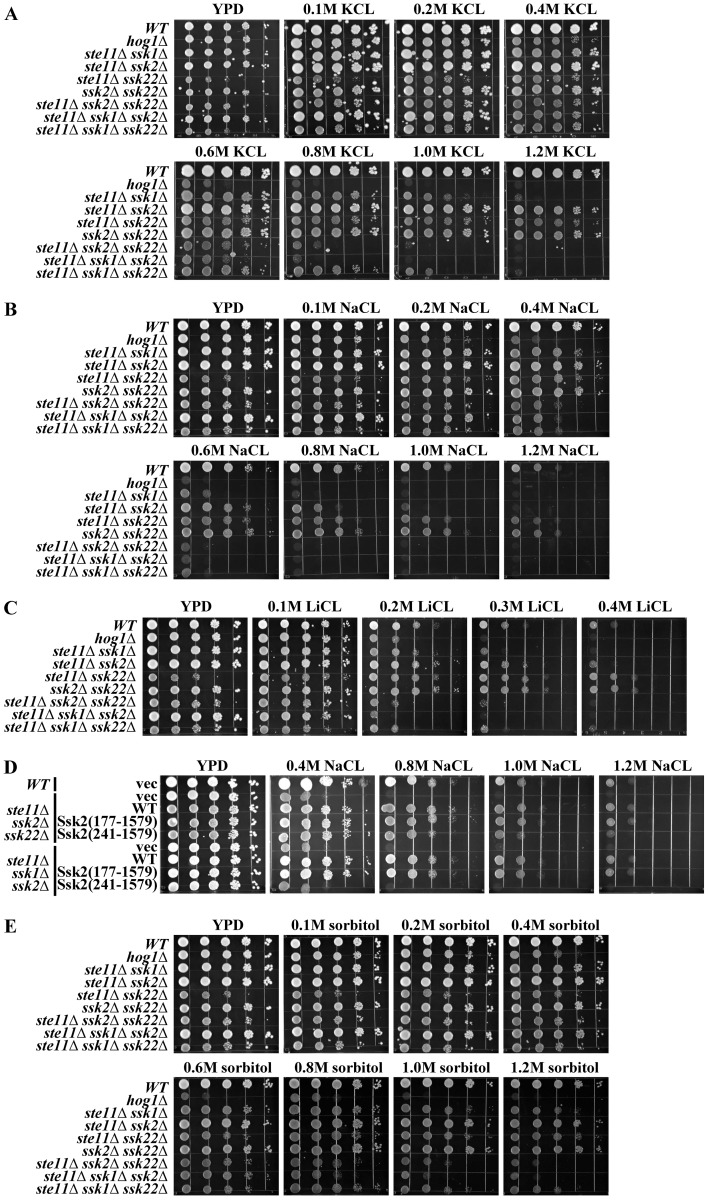
Ssk2p plays essential role in salt tolerance. A. The osmosensitivity phenotype of the *S. cerevisiae* HOG pathway mutants under KCL stress. B. The sodium-resistance phenotype of the *S. cerevisiae* HOG pathway mutants under various concentrations of NaCL. C. The lithium-sensitivity phenotype of the *S. cerevisiae* HOG pathway mutants under lithium stress. D. The truncated Ssk2 missing the amino acids 177∼240aa caused reduced salt resistance of the *ste11Δssk22Δ* cells. E. The osmosensitivity phenotype of the *S. cerevisiae* HOG pathway mutants under sorbitol stress. F. The phenotype of Ssk2 *Δ* ^(177∼239)^ cells is similar to that of the Ssk2*Δ* ^(1∼240)^ cells.

The yeast cells which grow in the presence of high sodium concentrations (salt stress) face both an elevated external osmotic environment and an increasing amount of Na^+^ entering the cells [37]. We have conducted a series of growth assay studies for the wild type and mutant cells under various levels of salt stress, with the results presented in Figure 6. The mutant *ssk2Δssk22Δ*, *ste11Δssk2Δ* and *ste11Δssk22Δ* showed no growth defect under severe osmotic stress (1.2 M sorbitol and 1.2 M KCL) (Figures 6A, 6E). However, the mutant *ste11Δssk2Δ* showed poorer growth when exposed to the poison level of cation (0.8 M NaCL and 0.3 M LiCL), which indicates that Ssk2p and Ste11p are essential for salt-tolerance (Figure 6A, 6B, and 6C). Actually, the mutant *ste11Δssk2Δ* grows as well as the wild type strain even when being exposed to 1.2 M sorbitol or 1.2 M KCL (Figures 6A and 6E).

The mutant *ste11Δssk1Δ* also displayed severe growth defect upon sodium stress, even the phosphorylation level of Hog1p under osmotic stress caused by NaCL was similar or slightly higher than that caused by the sorbitol or KCL (Figures 1A, 1D, 1F and 6B). The results imply that for salt tolerance, not only activation of Hog1p is required but MAPKKKs Ste11p and Ssk2p also play an important role. Although Ssk2p and Ssk22p are highly homologous, the Ssk2p shows better salt-tolerance than Ssk22p.

Furthermore, high level activation of Ssk2p is also required for the salt tolerance. As we discussed above, X factor can activate Ssk2p independent of Ssk1p and enhance the activation of Ssk2p by Ssk2p under osmotic stress. Here we found that the level of osmoresistance is slightly different between wild type Ssk2p cells and Ssk2*Δ* ^(1∼240)^ cells (Figure 6D). Lacking the binding site (amino acid 177∼240aa) for the X factor of Ssk2p would reduce the salt-resistance of the *ste11Δssk22Δ* cells (Figure 6D). The results indicate that the high level activation of Ssk2p is essential for saline-resistance.

## Discussion

It is well known that dephosphorylated Ssk1p can activate the Ssk2/Ssk22-Pbs2-Hog1 MAPK cascade. Though some studies indicated an additional input for Pbs2 [24], [25], there is no specific research on it. In this paper, we showed that Ssk2p can be activated and it then activates the HOG pathway independent of Ssk1p under osmotic stress. We propose that there is another regulator that can bind to the Ssk2p and activate the Ssk2p. The region which is essential for Ssk1p-independent activation of the HOG pathway is identified from aa 177 to aa 240 in Ssk2p. The findings can explain previous reports that *STL1* and *GRE2* are induced 8- to 38-fold in *ste11Δssk22Δ* cells but exhibit little induction (<1.7- fold) in *hog1Δ* or *pbs2Δ* strains [24].

We observed that deletion of the binding domain for the X factor significantly attenuates the activation of Hog1p under osmotic stress. It is possible that the deletion might change the conformation of Ssk2p, making it less accessible for Ssk1p. The observation also raised the possibility that binding of the unknown X factor increases the affinity of Ssk1p to Ssk2p in the presence of osmotic stress.

Budding yeast keeps three MAPKKKs, Ste11p, Ssk2p and Ssk22p, to activate one MAPKK Pbs2p to activate the HOG pathway upon hyperosmotic stress.At a crude level, they appear to be functionally redundant. However, as our study shows, they have distinct activation patterns. The Ste11 branch is less sensitive than the Sln1-Ssk1-Ssk2 cascade under mild osmotic shock, but it alone enables osmoresistance of the host cell almost as good as the wild type strain. The Sln1-Ssk1-Ssk2 cascade exhibit both sensitivity and tolerance to the various levels of osmotic stress. The X-Ssk2 branch only responds to the severe osmotic stress (i.e. at concentrations higher than 0.5 M sorbitol, KCL or NaCL). Its duration of activation is also much shorter. In comparison, the Sln1-Ssk1-Ssk22 cascade displays less sensitivity, slower activation, and low level of activation capacity even though Ssk22p is highly homologous to Ssk2p. The wild type cells employ a combination of these activation patterns in their osmostress response.

Besides the activation pattern, the three MAPKKKs in the HOG pathway have different roles in salt tolerance. Our study shows that Ste11p and Ssk2p cope with salt stress caused by sodium equally well, but Ssk22p displays a poorer capacity, implicating the role of Ste11p and Ssk2p in the activation of parallel processes when the cell is under toxic cation stress. Our results also show that the salt-resistance requires high level activation of Ssk2p, which could be achieved through synergistic activation of Ssk1p and the X factor.

In conclusion, we uncovered another input into Ssk2p in the HOG pathway and identified the receiver domain (amino acids 177∼240) in Ssk2p which is essential for the alternative activation pathway. Ssk2p is essential in salt tolerance besides its role in the activation of the HOG pathway. It would be very interesting if the experimental observations reported here can be followed up by protein structure studies to reveal the true binding domains and activation sites on the protein.
